# The migration law of magnesium ions during freezing and melting processes

**DOI:** 10.1007/s11356-021-17809-4

**Published:** 2021-12-02

**Authors:** Zhang Yan, Liu Tongshuai, Tang Yuanqing, Zhao Wanli, Ren Fangyun, Zhao Tongguo, Liu Yucan

**Affiliations:** College of Civil Engineering, Yantai University, Yantai, 264000 China

**Keywords:** Freezing and melting, Mg^2+^; Migration law, Distribution coefficient “K”, First principles, Exponential decay

## Abstract

To explore the migration law of magnesium ions (Mg^2+^) during freezing and melting processes, laboratory simulation experiments involving freezing and melting were carried out to investigate the influence of ice thickness, freezing temperature, initial concentration, and initial pH on the distribution of Mg^2+^ in the ice-water system. The distribution coefficient “K” (the ratio of the Mg^2+^ concentration in the ice layer to the Mg^2+^ concentration in the water layer under ice) was used to characterize the migration ability of Mg^2+^. The results showed that during the freezing process, the concentration distribution of Mg^2+^ in the ice and water two-phase system was as follows: ice layer < water before freezing < water layer under ice; in other words, it migrated from ice layer to the water layer under ice. “K” decreased with increasing ice thickness, freezing temperature, initial concentration, and initial pH; the higher the ice thickness, freezing temperature, initial concentration, and initial pH were, the higher the migration efficiency of Mg^2+^ into the water layer under ice was. During the melting process, Mg^2+^ was released in large amounts (50–60%) at the initial stage (0–25%) and in small amounts (25–100%) uniformly in the middle and later periods. According to the change of Mg^2+^ concentration in ice melt water, an exponential model was established to predict Mg^2+^ concentration in ice melt period. The migration law of Mg^2+^during the freezing and melting process was explained by using first principles.

**Figure:**
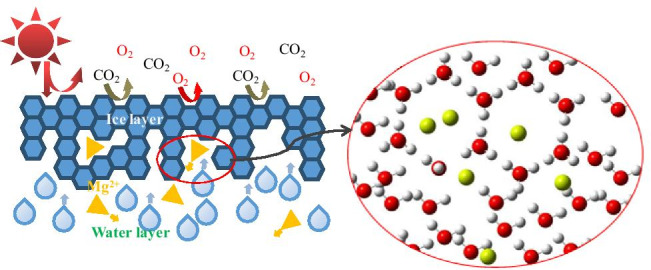
Graphical abstract

Graphical abstract

## Introduction

Icebound is an important hydrological feature of surface water at high latitudes. More than 50 million lakes regularly freeze every year in the world, and the annual icebound time is more than 150 days (Verpoorter et al. 2014). During the icebound period, the penetration rate of light in water is weakened (Catalan 1992; Welch et al. 1987), which affects the photosynthesis of aquatic plants under the ice (Jewson et al. 2009). In addition, due to the ice sheet, the oxygen exchange process between the water layer under ice and the atmosphere is hindered, and the metabolism of aquatic organisms is affected; as a result, the self-purification capacity of the water layer is greatly reduced (Kirillin et al. 2012). Therefore, the environment of the water layer under ice cover has its own particularity.

Among the published literature on fresh water, only 2% involves the freezing process of water bodies (Hampton et al. 2015), and these studies mainly focus on heavy metals and nutrients. Pieters and Lawrence (2009) found in winter that in the Tailings Lake in northwest Canada, when the ice thickness reached 60–80 cm, approximately 99% of the salt in the ice was discharged into the water layer under ice. Liu et al. (2017, 2019) found that during the growth period of sea ice in Lake Ulansuhai, due to the difference in equilibrium gradient, Hg, Zn, and Pb concentrations in water first increased during the freezing process; then the dynamic balance of ions between water and sediments was disturbed; and a portion of the Hg, Zn, and Pb concentrations migrated to sediments. Melak et al. (2016) found that in the Rift Valley Lake in Ethiopia in winter, Cr (VI) content ranged from 0.104 to 0.121 mg·L^−1^, which exceeded the drinking water standard of Ethiopia and WHO by 0.05 mg·L^−1^. Hampton et al. (2015) conducted the first global quantitative synthesis of 101 lakes and found that the total dissolved nitrogen and total nitrogen were higher in the water layer during the icebound period. Li et al. (2014) found that nutrient salts and algae continuously migrated to the water layer under ice in Lake Ulansuhai during the icebound period, leading to intensified eutrophication of the water layer under ice. Shafique et al. (2016) found that in the process of directional freezing of water layer, gas will also migrate downward with the growth of ice front. In addition, during the freezing process, some pollutants in the water were captured in the ice sheet, and in the spring, when it melted, the pollutants were released into the water environment within a short time, bringing great impacts to the aquatic ecosystem in early spring (Chiou et al. 1986; Huang et al. 2009, 2012).

Although there have been relevant studies on the migration law of heavy metals and organic matter in the process of icing, there are relatively few studies on the migration law and mechanism of inorganic ions in the process of ice sealing. As we all know, the lake water contains a variety of inorganic salts, such as Li^+^, K^+^, Mg^2+^/Cl^−^, and SO_4_^2−^ (Sun et al.^1995^). Magnesium is one of the major elements in lake water, and the content of Mg^2+^ in salt lakes is more abundant. The Dead Sea has the highest Mg^2+^ content, up to 190 g·L^−1^, with a total reserve of 230 × 108 t (Wisniak 2002), followed by the Great Salt Lake in the USA, with a content of 6 × 108 t. During the ice sealing period, Mg^2+^ are discharged from the ice layer and then enter the water layer under ice, resulting in the concentration of Mg^2+^ in the water layer under ice. With the progress of icing process, the concentration of Mg^2+^ in the water layer under ice increases gradually (Cui et al. 2013). The continuous increase of Mg^2+^ concentration will affect the pH of sediments and endanger the ecological health of lakes (Chen et al. 2008; Dai 2016). In the process of ice melting, if the early concentrated release of Mg^2+^ in ice layer will also increase the concentration of Mg^2+^ in the water layer under ice. If it is used as source water, the treatment effect of the original treatment process is poor compared with that before freezing, which is easy to cause the increase of Mg^2+^ concentration in drinking water. The high concentration of Mg^2+^ in drinking water will affect the absorption of calcium ions. When the concentration of Mg^2+^ in the blood is higher than 1.0 mg·L^−1^, it will lead to chronic hypermagnesemia (Musso 2009), and continuous hypermagnesemia will lead to chronic kidney disease (Guan 2017).

Therefore, this study took Mg^2+^ as the research object to carry out simulation experiments, which purpose was to explore the distribution law of Mg^2+^ in the ice water system during the freezing and melting process and investigate the influence of ice thickness, freezing temperature, initial concentration, and initial pH on the distribution of Mg^2+^ in the ice water phase. The distribution coefficient “K” was used to characterize the Mg^2+^ migration ability. In addition, we discussed the release law of Mg^2+^ during the melting process. The above laws were explained from first principles. We hope that this study can draw researchers’ attention to the changes in water quality under ice during the icebound period.

## Experimental section

### Experimental setup

**Fig. 1:**
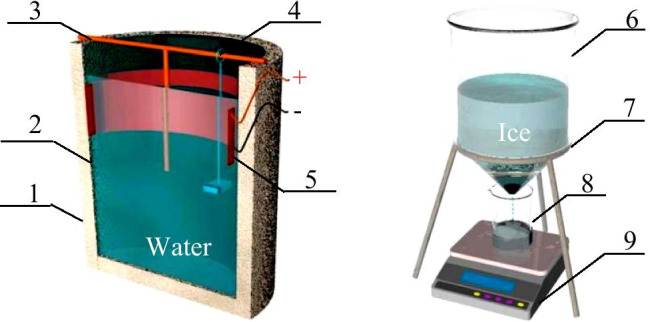
Schematic diagram of freeze-melt experimental device: on the left is the freezing device; on the right is the ice melt device (1 EPS insulation; 2 glass cylindrical barrel; 3 T-shaped bracket; 4 measuring ice thickness device; 5 heating sheet; 6 ice melting funnel; 7 funnel support; 8 beaker; 9 electronic balance)

In order to simulate the top-down directional freezing process of natural water, an open unidirectional freezing simulation device (Fig. 1) was designed to realize the vertical slow freezing of solution. The upper part of the device had an opening for one-way transfer of cold energy and a built-in reactor (wall thickness 5 mm, outer diameter 20 cm, height 37.5 cm) with high borosilicate glass. Around and at the bottom of the glass were wrapped with EPS (expanded polystyrene) insulation material to block the heat transfer between the reactor and outside. To facilitate icicle removal, a temperature-controlled heating sheet was wrapped between the outer wall of the reactor and insulation material, and a resistance wire (nickel–chromium alloy) thickness measuring device was placed in the barrel; the resistance wire displacement difference indicates the ice thickness. The above devices were placed in a chest freezer (BC/BD-519 HEX, Haier, Qingdao, China) with a volume of 519 L and a minimum refrigeration temperature of -40℃.

The ice melt device was a self-made stainless steel funnel (Fig. 1). The ice layer was stored in the upper part, the beaker was placed in the lower part, and the electronic balance was placed under the beaker to weigh the ice melt-water.

### Experimental design and methods

To explore the migration law of Mg^2+^ in the water freezing process, as well as the influence of ice thickness, freezing temperature, initial concentration, and initial pH on the migration law, the experimental design was as follows:

- (1) To study the effect of ice thickness on the migration law of Mg^2+^ in the freezing process, a standard solution of Mg^2+^ with a concentration of 500 mg·L^−1^ was prepared and placed in five freezing simulators. Then, an 8-L water sample with pH 7 was put into each simulator, the height of water was 28 cm, and devices were placed in a low-temperature box at – 15 ℃. When the ice thickness reached 4 cm, 8 cm, 12 cm, 16 cm, and 20 cm; the reactors were removed.
- (2) To study the effects of freezing temperature on the migration law of Mg^2+^ in the freezing process, a standard solution of Mg^2+^ with a concentration of 500 mg·L^−1^ was prepared and placed in five freezing simulators. Then, an 8-L water sample with pH 7 was put into each simulator, the height of water was 28 cm, and devices were placed in a low temperature box at – 5 ℃,—10 ℃,—15 ℃,—20 ℃, and—25℃. When the ice thickness reached 12 cm, the reactors were removed.
- (3) To study the effects of initial concentration on the migration law of Mg^2+^ in the freezing process, referring to *Guidelines for Drinking-water Quality* set by the WHO (2011), the maximum allowable level of total hardness (calculated by CaCO_3_) of drinking water shall not exceed 500 mg·L^−1^. The standard solutions of Mg^2+^ with concentrations of 300 mg·L^−1^, 400 mg·L^−1^, 500 mg·L^−1^, 600 mg·L^−1^, and 700 mg·L^−1^ were placed in five freezing simulators. Then, an 8-L water sample with pH 7 was placed into each simulator, the height of water was 28 cm, and devices were placed in a low-temperature box at – 15 ℃. When the ice thickness reached 12 cm; the reactors were removed.
- (4) To study the effects of initial pH on the migration law of Mg^2+^ in the freezing process, a standard solution of Mg^2+^ with a concentration of 500 mg·L^−1^ was prepared and placed in five freezing simulators. Then, an 8-L water sample was put into each simulator; the height of water was 28 cm; and the pH of the water samples were adjusted to 5.5, 6.5, 7.5, 8.5, and 9.5 by dropping HCl and NaOH solution and placing devices in a low temperature box at – 15 °C. When the ice thickness reached 12 cm, the reactors were removed.

The ice sample obtained from the above freezing experiment was put into a beaker, melted in a constant temperature box (25 °C), and the water under the ice was evenly mixed and put into a beaker for detecting.

The process of ice samples acquisition in the melting experiment was the same as that in the simulated freezing experiment. The ice samples with different ice thickness, freezing temperature, initial concentration, and initial pH are weighed, placed in the ice-melt device, and melted in the incubator (25 °C). Take the ice melt water four times on average according to the ice sample weight, and the weight of each melt water accounts for 25% of the total weight of the ice sample. The melt water weight reaches 25% of the total weight of ice sample for the first time as melting 1, 25% of the total weight of ice sample for the second time as melting 2, 25% of the total weight of ice sample for the third time as melting 3, and 25% of the total weight of ice sample for the fourth time as melting 4. The concentrations of Mg^2+^ were detected respectively.

### Sample detection methods

The Mg^2+^ concentrations in ice melt water and the water layer under ice were measured in accordance with the *Water and Wastewater Monitoring Analysis Method* (4th ed.) (2002), and the standard deviation of the detection results was controlled within 5%. The generated solid was observed by optical microscope with an XST-107 T Digital Microscope, which can achieve a magnification of up to 1000 times.

### Data analysis methods

The distribution coefficient “K” is defined as the ratio between the average concentration of Mg^2+^ in the ice layer and the average concentration of Mg^2+^ in the water layer under ice, which reflects the migration ability and discharge effect of Mg^2+^ in the process of water freezing:

1 $$K=\frac{{C}_{i}}{{C}_{w}}$$

where ${C}_{i}$ is the average concentration of Mg^2+^ in the ice layer and ${C}_{w}$ is the average concentration of Mg^2+^ in the water layer under ice (mg·L^−1^).

## Results

### Migration law of Mg^2+^ during the freezing process

#### Migration of Mg^2+^ in the ice and water two-phase system under different ice thicknesses conditions

**Fig. 2:**
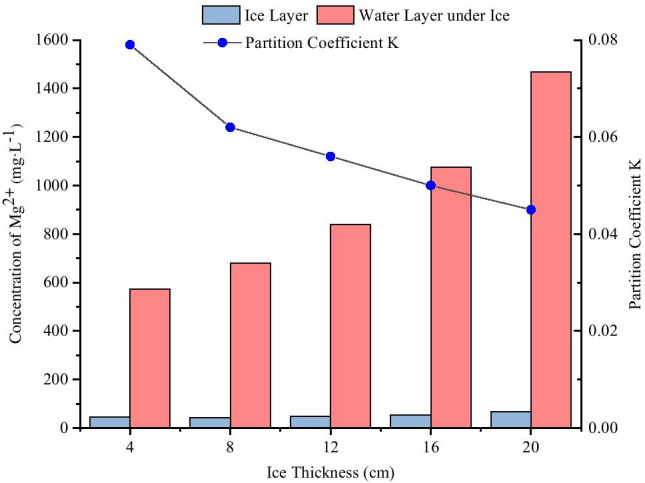
Distributions of Mg^2+^ in the ice and water two-phase system with different ice thicknesses

The ice melt water and water layer under ice obtained from different ice thickness conditions were collected, and the concentrations of Mg^2+^ were measured (Table 1). The distributions of Mg^2+^ in the ice and water two-phase under different ice thickness conditions are shown in Fig. 2:

**Table 1:** Concentrations of Mg^2+^ in ice and water system from different icing thicknesses

Ice thickness (cm)	Freezing temperature (°C)	Initial concentration(mg·L^−1^)	pH	Concentration in ice layer (mg·L^−1^)	Concentration of water layer under ice (mg·L^−1^)	K
4	-15	500	7	45.458	573.08	0.079
8	-15	500	7	42.461	681.276	0.062
12	-15	500	7	46.953	838.829	0.056
16	-15	500	7	53.276	1075.645	0.050
20	-15	500	7	66.477	1468.248	0.045

As shown in Fig. 2, when the ice thicknesses were 4 cm, 8 cm, 12 cm, 16 cm, and 20 cm, the concentrations of Mg^2+^ in ice layer were significantly lower than that of raw water by 500 mg·L^−1^, while the concentrations of Mg^2+^ in the water layer under ice were significantly higher than that of raw water by 500 mg·L^−1^. The concentrations of Mg^2+^ in ice layer were 0.091, 0.085, 0.094, 0.107, and 0.133 times of the initial concentration respectively, and the concentrations of Mg^2+^ in the water layer under ice were 1.146, 1.363, 1.678, 2.151, and 2.936 times of the initial concentration respectively. The results showed that the higher the ice thickness, the higher the concentration of Mg^2+^ in the ice layer, and the higher the concentration of Mg^2+^ in the water layer under ice. With increased ice thickness, the distribution coefficient “K” decreased. In other words, with increased ice thickness, the ability of Mg^2+^ to migrate into the water layer under ice became stronger.

#### Migration of Mg^2+^ in the ice and water two-phase system under different freezing temperature conditions

**Fig. 3:**
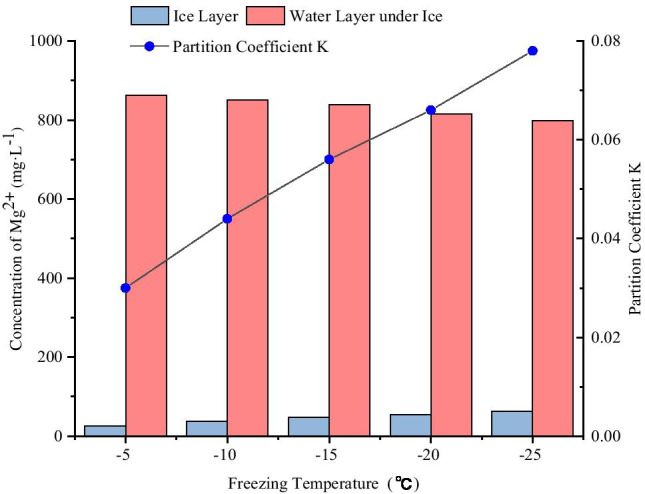
Distributions of Mg^2+^ in the ice and water two-phase system with different freezing temperatures

The ice melt-water and the water layer under ice obtained from different freezing temperature conditions were collected, and the concentrations of Mg^2+^ were measured (Table 2). The distributions of Mg^2+^ in the ice and water two-phase system under different freezing temperature conditions are shown in Fig. 3.

**Table 2:** Concentrations of Mg^2+^ in ice and water system from different freezing temperatures

Freezing temperature (°C)	Ice thickness (cm)	Initial concentration(mg·L^−1^)	pH	Concentration in ice layer (mg·L^−1^)	Concentration of water layer under ice (mg·L^−1^)	K
-5	12	500	7	26.077	862.516	0.030
-10	12	500	7	37.771	849.896	0.044
-15	12	500	7	46.978	838.829	0.056
-20	12	500	7	53.919	815.148	0.066
-25	12	500	7	62.261	798.827	0.078

As shown in Fig. 3, when the freezing temperatures were – 5 ℃,—10 ℃,—15 ℃,—20 ℃, and – 25 ℃, the concentrations of Mg^2+^ in ice layer were significantly lower than that of raw water by 500 mg·L^−1^, while the concentrations of Mg^2+^ in the water layer under ice were significantly higher than that of raw water by 500 mg·L^−1^. The concentrations of Mg^2+^ in ice layer were 0.052, 0.076, 0.094, 0.108, and 0.125 times of the initial concentration respectively, and the concentrations of Mg^2+^ in the water layer under ice were 1.73, 1.70, 1.68, 1.63, and 1.60 times of the initial concentration respectively. Hence, the observation was the lower the freezing temperature, the higher the concentration of Mg^2+^ in the ice layer, and the lower the concentration of Mg^2+^ in the water layer under ice. With decreased freezing temperature, the distribution coefficient “K” increased. In other words, with decreased freezing temperature, the ability of Mg^2+^ to migrate into the water layer under ice became weaker.

#### Migration of Mg^2+^ in the ice and water two-phase system under different initial concentration conditions

**Fig. 4:**
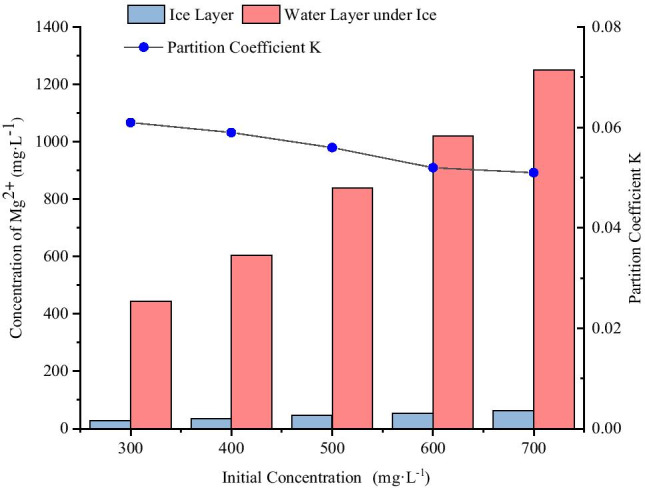
Distributions of Mg^2+^ in the ice and water two-phase system with different initial concentrations

The ice melt water and water layer under ice obtained from different initial concentration conditions were collected, and the concentrations of Mg^2+^ were measured (Table 3). The distributions of Mg^2+^ in the ice and water two-phase system under different initial concentration conditions are shown in Fig. 4.

**Table 3:** Concentrations of Mg^2+^ in ice and water system from different initial concentrations

Initial concentration (mg·L^−1^)	Ice thickness (cm)	Freezing temperature (°C)	pH	Concentration in ice layer (mg·L^−1^)	Concentration of water layer under ice (mg·L^−1^)	K
300	12	-15	7	26.876	443.851	0.061
400	12	-15	7	35.750	603.574	0.059
500	12	-15	7	46.978	838.829	0.056
600	12	-15	7	53.455	1020.465	0.052
700	12	-15	7	63.879	1250.852	0.051

As shown in Fig. 4, when the concentrations of Mg^2+^ standard solution were 300 mg·L^−1^, 400 mg·L^−1^, 500 mg·L^−1^, 600 mg·L^−1^, and 700 mg·L^−1^, the concentrations of Mg^2+^ in ice layer were significantly lower than that of raw water, while the concentrations of Mg^2+^ in the water layer under ice were significantly higher than that of raw water. The concentrations of Mg^2+^ in ice layer were 0.054, 0.072, 0.094, 0.107, and 0.128 times of the initial concentrations respectively, and the concentrations of Mg^2+^ in the water layer under ice were 1.480, 1.509, 1.678, 1.701, and 1.787 times of the initial concentration respectively. The results showed that the higher the initial concentration, the higher the concentration of Mg^2+^ in the ice layer, and the higher the concentration of Mg^2+^ in the water layer under ice. With increased initial concentration, the distribution coefficient “K” decreased. In other words, with increased initial concentration, the ability of Mg^2+^ to migrate into the water layer under ice became stronger.

#### Migration of Mg^2+^ in the ice and water two-phase system under different initial pH conditions

**Fig. 5:**
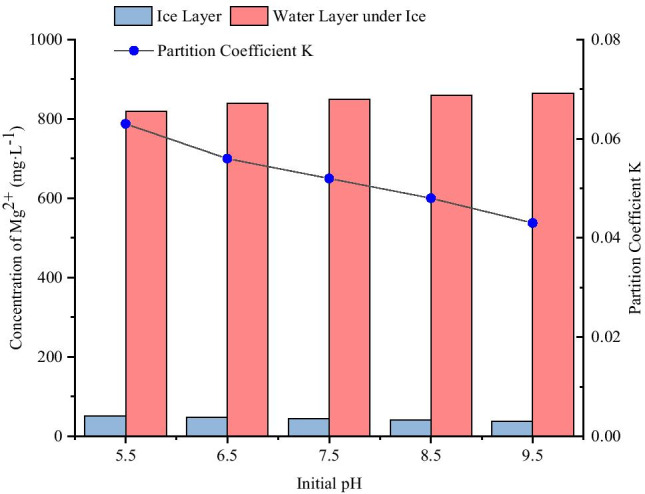
Distributions of Mg^2+^ in the ice and water two-phase system with different initial pH

The ice melt water and water layer under ice obtained from different initial pH conditions were collected, and the concentrations of Mg^2+^ were measured (Table 4). The distributions of Mg^2+^ in the ice and water two-phase system under different initial pH conditions are shown in Fig. 5.

**Table 4:** Concentrations of Mg^2+^ in ice and water system from different initial pH

pH	Ice thickness (cm)	Freezing temperature (°C)	Initial concentration (mg·L^−1^)	Concentration in ice layer (mg·L^−1^)	Concentration of water layer under ice (mg·L^−1^)	K
5.5	12	-15	500	51.586	819.873	0.063
6.5	12	-15	500	46.953	838.829	0.056
7.5	12	-15	500	44.213	849.652	0.052
8.5	12	-15	500	41.210	858.745	0.048
9.5	12	-15	500	37.368	864.785	0.043

As shown in Fig. 5, when the pH of magnesium standard solution were 5.5, 6.5, 7.5, 8.5, and 9.5, the concentrations of Mg^2+^ in ice layer were significantly lower than that of raw water by 500 mg·L^−1^, while the concentrations of Mg^2+^ in the water layer under ice were significantly higher than that of raw water by 500 mg·L^−1^. The concentrations of Mg^2+^ in ice layer were 0.103, 0.094, 0.088, 0.082, and 0.075 times of the initial concentration, respectively, and the concentrations of Mg^2+^ in the water layer under ice were 1.640, 1.678, 1.699, 1.717, and 1.730 times of the initial concentration respectively. The results showed that the higher the initial pH, the lower the concentration of Mg^2+^ in the ice layer, and the higher the concentration of Mg^2+^ in the water layer under ice. With increased initial pH, the distribution coefficient “K” decreased. In other words, with increased initial pH, the ability of Mg^2+^ to migrate into the water layer under ice became stronger.

### Release law of Mg^2+^ during ice melting

#### Release law of Mg^2+^ in the melting process of ice layers under different ice thicknesses

**Fig. 6:**
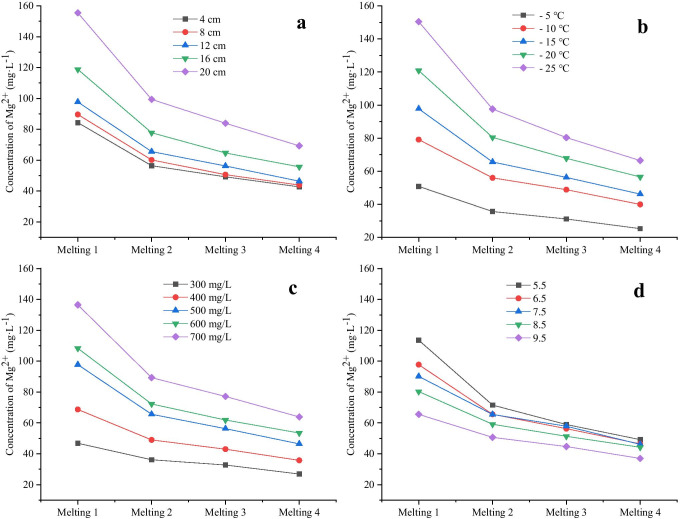
Release law of Mg^2+^ during the ice melting process under different freezing conditions. **a** Ice thickness, **b** freezing temperature, **c** initial concentration, **d** initial pH

As shown in Fig. 6a, for melting 1 of ice samples obtained from different ice thicknesses (4 cm, 8 cm, 12 cm, 16 cm, and 20 cm), the concentrations of Mg^2+^ were 84.375 mg·L^−1^, 89.634 mg·L^−1^, 97.745 mg·L^−1^, 118.856 mg·L^−1^, and 155.48 mg·L^−1^, respectively, which were 1.97–2.24 times the average concentration of Mg^2+^ in the ice layer. The ratios of Mg^2+^ concentration in melting 1 to Mg^2+^ concentration in corresponding ice layer were 49.36%, 51.06%, 52.64%, 53.34%, and 56.07%, respectively. For meltings 2–4 of ice samples obtained from different ice thicknesses, the average concentrations of Mg^2+^ were 28.854 mg·L^−1^, 28.641 mg·L^−1^, 29.31 mg·L^−1^, 34.664 mg·L^−1^, and 40.601 mg·L^−1^, respectively, which were 0.58–0.68 times of the average concentration of Mg^2+^ in the ice layer. The ratios of the average Mg^2+^ concentration in meltings 2–4 to the Mg^2+^ concentration in the corresponding ice layer were 50.64%, 48.94%, 47.36%, 46.66%, and 43.93%, respectively.

#### Release law of Mg^2+^ in the melting process of ice layers under different freezing temperatures

As shown in Fig. 6b, for melting 1 of ice samples obtained from different freezing temperatures (- 5 ℃,—10 ℃,—15 ℃,—20 ℃,—25 ℃), the concentrations of Mg^2+^ were 50.851 mg·L^−1^, 79.125 mg·L^−1^, 97.745 mg·L^−1^, 120.852 mg·L^−1^, and 150.452 mg·L^−1^, respectively, which were 1.98–2.26 times of the average concentration of Mg^2+^ in the ice layer. The ratios of Mg^2+^ concentration in melting 1 to Mg^2+^ concentration in corresponding ice layer were 50.29%, 49.53%, 52.93%, 53.45%, and 56.60%, respectively. For meltings 2–4 of ice samples obtained from different freezing temperatures, the average concentrations of Mg^2+^ were 16.752 mg·L^−1^, 26.879 mg·L^−1^, 28.98 mg·L^−1^, 35.114 mg·L^−1^, and 38.452 mg·L^−1^, respectively, which were 0.57–0.67 times of the average concentration of Mg^2+^ in the ice layer. The ratios of the average Mg^2+^ concentration in meltings 2–4 to the Mg^2+^ concentration in the corresponding ice layer were 49.71%, 50.47%, 47.07%, 46.55%, and 43.4%, respectively.

#### Release law of Mg^2+^ in the melting process of ice layers under different initial concentrations

As shown in Fig. 6c, for melting 1 of ice samples obtained from different initial concentrations (300 mg·L^−1^, 400 mg·L^−1^, 500 mg·L^−1^, 600 mg·L^−1^, 700 mg·L^−1^), the concentrations of Mg^2+^ were 46.838 mg·L^−1^, 68.734 mg·L^−1^, 97.745 mg·L^−1^, 108.335 mg·L^−1^, and 136.476 mg·L^−1^, respectively, which were 1.74–2.14 times of the average concentration of Mg^2+^ in the ice layer. The ratios of Mg^2+^ concentration in melting 1 to Mg^2+^ concentration in corresponding ice layer were 43.57%, 48.06%, 52.64%, 50.67%, and 53.41%, respectively. For meltings 2–4 of ice samples with different initial concentration, the average concentrations of Mg^2+^ were 20.222 mg·L^−1^, 24.772 mg·L^−1^, 29.310 mg·L^−1^, 35.162 mg·L^−1^, and 39.680 mg·L^−1^, respectively, which were 0.62–0.75 times of the average concentration of Mg^2+^ in the ice layer. The ratios of the average Mg^2+^ concentration in meltings 2–4 to the Mg^2+^ concentration in the corresponding ice layer were 56.43%, 51.94%, 47.36%, 49.33%, and 46.59%, respectively*.*

#### Release law of Mg^2+^ in the melting process of ice layers under different initial pH

As shown in Fig. 6d, for melting 1 of ice samples obtained from different initial pH (5.5, 6.5, 7.5, 8.5, 9.5), the concentrations of Mg^2+^ were 113.596 mg·L^−1^, 97.745 mg·L^−1^, 90.125 mg·L^−1^, 80.29 mg·L^−1^, and 65.523 mg·L^−1^, respectively, which were 1.77–2.31 times of the average concentration of Mg^2+^ in the ice layer. The ratios of Mg^2+^ concentration in melting 1 to Mg^2+^ concentration in corresponding ice layer were 59.01%, 54.69%, 52.64%, 48.79%, and 44.59%, respectively. For meltings 2–4 of ice samples with different initial pH, the average concentrations of Mg^2+^ were 27.696 mg·L^−1^, 29.310 mg·L^−1^, 31.422 mg·L^−1^, 32.171 mg·L^−1^, and 27.497 mg·L^−1^, respectively, which were 0.55–0.74 times of the average concentration of Mg^2+^ in the ice layer. The ratios of the average Mg^2+^ concentration in meltings 2–4 to the Mg^2+^ concentration in the corresponding ice layer were 40.99%, 45.31%, 47.36%, 51.21%, and 55.41%, respectively.

## Analysis and discussion

### Migration mechanism of Mg^2+^ during the freezing process

According to *Guidelines for Drinking-water Quality* set by the WHO (2011), the maximum allowable level of total hardness (calculated by detecting CaCO_3_) of drinking water shall not exceed 500 mg·L^−1^. In the simulated freezing experiment, the detection range of Mg^2+^ in ice layers was 26.077–66.477 mg·L^−1^ when the concentration of Mg^2+^ was 500 mg·L^−1^, which is lower than the limit value of hardness in *the Guidelines for Drinking-water Quality*, while the detection range of Mg^2+^ in the water layers under ice was 573.08–1468.248 mg·L^−1^, which is significantly higher than the initial concentration of 500 mg·L^−1^ and far beyond the limit value of hardness in *the Guidelines for Drinking-water Quality*. Under different conditions, the concentration relationship of Mg^2+^ in the ice and water two-phase system is as follows: ice layer < water layer before freezing < water layer under ice; in other words, Mg^2+^ migrate from the ice layer to the water layer under ice during the freezing process. The migration process can be explained by first principles.

**Fig. 7:**
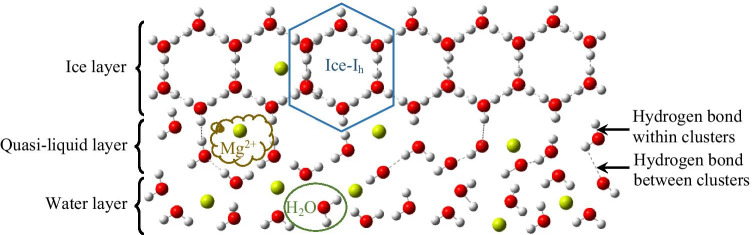
Schematic diagram of Mg^2+^ migration in the ice and water two-phase system

From the microscopic point of view, in the unfrozen state, water molecules are relatively free, and Mg^2+^ in water are tightly surrounded by water molecules(Wang et al. 2010).When the temperature decreases, energy decreases; water molecules and Mg^2+^ begin to move. At a certain time, the position structure of water molecules and Mg^2+^ appears to be optimal (Fig. 7), and the energy is optimal for the coexistence of water molecules and Mg^2+^. As the energy continues to decrease, water molecules begin to transform into tiny ice crystals. At this time, water molecules not only interact with Mg^2+^ but also form ice crystal structures, which result in insufficient free water molecules surrounding Mg^2+^. From the microscopic model, Mg^2+^ are more stable in the water phase than in the ice phase. Due to the tendency of ions to move towards the stable phase, Mg^2+^ will migrate from the unstable ice phase to the stable water phase in the ice and water two-phase system.

From a macroscopic point of view, when water begins to freeze, the free water molecules are transformed into regular ice structures, and the ice and water two-phase system appears in the solution. The energy that must be overcome by Mg^2+^ in combination with the ice phase is small (Sun et al. 2018), so the energy released by Mg^2+^ in combination with the ice phase is small, and the energy of the whole system is high, so the system formed in the ice layer is unstable. The energy to be overcome by Mg^2+^ in combination with the water phase is large, so the energy released by Mg^2+^ in combination with the water phase is large, and the energy of the whole system is low, so the system formed in the water layer is relatively stable. Therefore, Mg^2+^ migrates from the ice layer to the water layer under ice.

### Effects of different factors on Mg^2+^ migration during the freezing process

In the simulated freezing experiment, the distribution coefficient “K” was negatively correlated with ice thickness, freezing temperature, initial concentration, and initial pH; in other words, the larger the ice thickness, freezing temperature, initial concentration, and initial pH, the smaller the “K” value and the stronger the migration ability of Mg^2+^ to the water layer under ice.

**Fig. 8:**
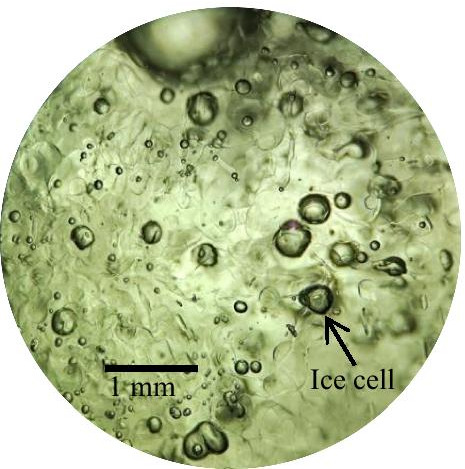
Ice cell structure. Freezing temperature of—15℃, 40 × magnification

The migration ability of Mg^2+^ in the ice and water two-phase system is related to hydrogen bonding, which can be divided into hydrogen bonds formed between water clusters and hydrogen bonds formed within water clusters according to different hydrogen bond positions (Fig. 7). There is a competitive relationship between them, and the role of hydrogen bonds within clusters is far greater than that of hydrogen bonds between clusters (Wang and Wang 2001). Pure ice has a tetrahedral crystal structure and is formed by water molecules associating through hydrogen bonds (Sun 2019). The association among molecules will result in the release of a large amount of energy, and the rate of energy release determines the growth rate of ice crystals. When the energy is low, the thermal motion of water molecules is relatively weak, and the formation rate of hydrogen bonds is accelerated; thus, the formation rate of ice crystals is accelerated. Since the temperature is greater than the solution of the substance diffusion, the solid–liquid interface is dendritic growth (Shum and Papangelakis 2019), and Mg^2+^ have no time to “escape” and are trapped in the dendritic space to form ice cells (such as Fig. 8). Generally, higher growth rates lead to more complex ramifications in ice crystals and result in narrow channels with higher streaming resistance and less ions expulsion (Breitner 1953). The freezing temperature and ice thickness affect the energy release rate because the increase in ice sheet thickness hinders the heat exchange between the water layer under ice and the outside world. When the temperature is higher and the ice thickness is larger, the growth rate of ice crystals is lower; in the case of slow growth, the size of each ice crystal does not change much, and Mg^2+^ and water molecules form a relatively balanced relationship between the ice and water two-phase system. The volume of newly formed ice crystals increases, but the ability to capture Mg^2+^ decreases (Okawa et al. 2009). Therefore, with increased ice thickness and freezing temperature, the distribution coefficient “K” of Mg^2+^ in the ice and water two-phase system decreases.

In addition, hydrogen bonds between water clusters also affect the migration ability of Mg^2+^in the ice and water two-phase system. For the hydrogen bonds formed between water clusters, when the solution concentration increases, the effect of hydrogen bonds between clusters increases (Wang et al. 2010), the diffusion coefficient decreases, more granular ice crystals are formed, and the surface area of ice crystals increases (Yu et al. 2007). Although more Mg^2+^ have no time to “escape” and be captured in the ice layer at the early stage of icing, with the increase in ice thickness, the crystal grows at a uniform speed and Mg^2+^ continue to migrate, and the increase of Mg^2+^ in the ice layer is much smaller than that in the water layer under ice, leading to a decreased “K” value. Similarly, the increase of pH can lead to a fundamental change in the structure of water clusters in Mg^2+^ solutions. With increased pH, the distance between Mg-O atoms decreases (Li et al. 2004), which makes the hydration number of Mg^2+^ in the electrolyte solution decrease (Kang et al. 2014); the “escape” rate of Mg^2+^ to the water is accelerated, which makes it difficult to capture with ice layer; and there are fewer Mg^2+^ left in the ice layer after freezing. Therefore, with the increase of initial concentration and initial pH, the distribution coefficient “K” of Mg^2+^ in the ice and water two-phase system decreases.

### Effects of different factors on Mg^2+^ release during ice melting

The results of ice melting experiments showed that the release law of Mg^2+^ from the ice phase was consistent with different conditions during the melting process: In the early stage of melting (0–25%), a large amount of Mg^2+^ were rapidly released in a short time. The concentration of Mg^2+^ in the initial melting stage was much higher than the average concentration of Mg^2+^ in the ice layer, and then the release rate of Mg^2+^ rapidly decreased, showing a small amount of uniform release phenomenon. The concentration of Mg^2+^ in the middle and late stages was significantly lower than the average concentration of Mg^2+^ in the ice layer.

**Fig. 9:**
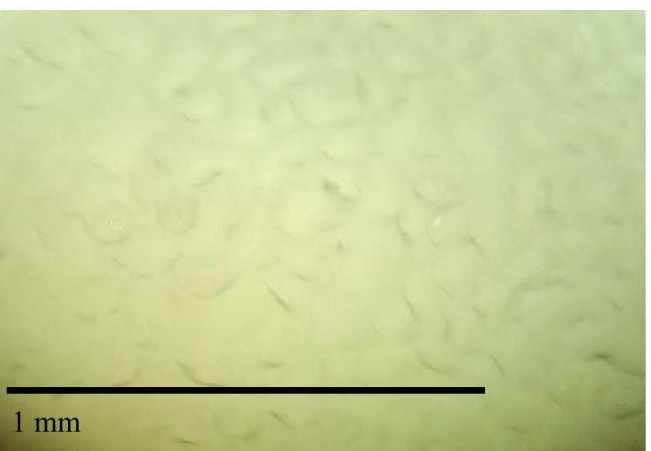
The surface of the solid continues to melt, 200 × magnification

**Fig. 10:**
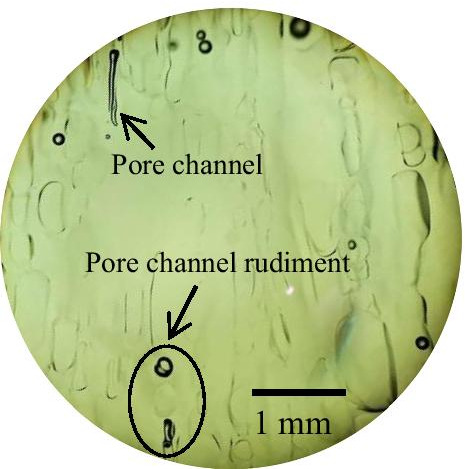
Ice channel structure. Freezing temperature of -15 °C, 40 × magnification

In the ice melting process, the surface ice begins to melt, and droplets form on the ice surface (such as Fig. 9). The melting rate depends on the heat transfer from the environment to the melting interface, and the melting rate of ice is equivalent to the growing rate of droplets on the melting interface (Nakagawa et al. 2010). The growth of droplets is a continuous process, and Mg^2+^ can be attached to the growing droplets, which is defined as “solute elution” (Shafique et al. 2012). “Solute elution” into droplets will be limited by the droplet growth rate. Therefore, the dynamic equilibrium between the droplet growth rate and Mg^2+^ diffusion rate determines the concentration of Mg^2+^ in the melting process. When the diffusion coefficient of Mg^2+^ is sufficiently large in the refrigerant liquid phase, the growth rate of droplets will affect the concentration level in the melting stage. In other words, slow droplet growth conditions are conducive to the recovery of solutes with high yields (Badawy 2016). In the early melting period, with the increase and interconnection of melting pores, many pore channels connecting the ice interior are formed (such as Fig. 10), and the ice layer becomes loose and disintegrates, resulting in the release of a large amount of Mg^2+^ in the early stage of ice melting. With ice layer melting, most Mg^2+^ have “escaped” out of the ice layer along the channel, leaving only a small amount of Mg^2+^ in ice layer. In the middle and later periods of melting, the release of Mg^2+^ shows a decreasing trend, and the number of Mg^2+^ is small and uniform.

### Exponential model of ice melting

**Fig. 11:**
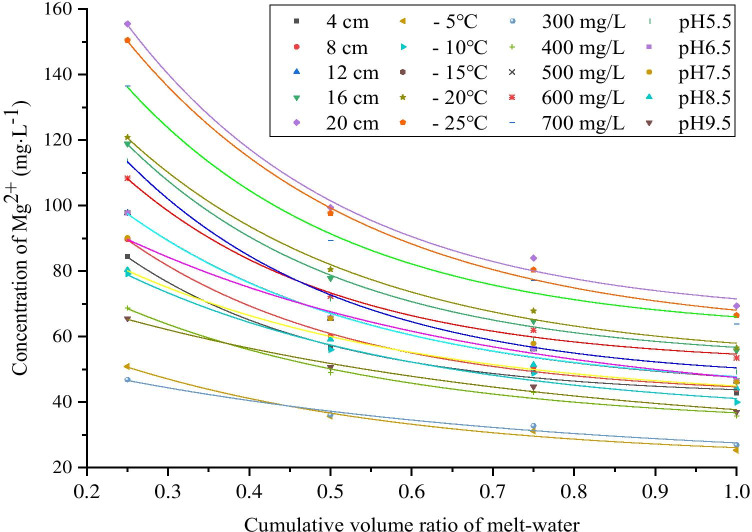
The fitting curves of the change in Mg^2+^ concentration under different freezing conditions

To quantitatively describe the release law of Mg^2+^ in melting, the origin is used to fit the data of melting water under various conditions. The results show that as the cumulative volume ratio of ice melt water increases, the ratio of Mg^2+^ concentration in ice melt water to the concentration of ice layer decreases, and the relationship between them decreases exponentially, as shown in Fig. 11. The fitting parameters are shown in Tables 5–8. The general formula is

**Table 5:** Parameter values under different ice thicknesses

Drawing	4 cm	8 cm	12 cm	16 cm	20 cm
*y*_*0*_	41.62 ± 3.43	41.42 ± 2.86	43.15 ± 6.48	52.61 ± 3.69	65.83 ± 8.32
*A*	115.43 ± 23.31	118.76 ± 14.15	123.36 ± 23.30	167.20 ± 19.92	223.85 ± 43.49
*t*	0.25 ± 0.06	0.28 ± 0.05	0.30 ± 0.10	0.27 ± 0.04	0.27 ± 0.07
*R*^2^ (coefficient of determination)	0.99	1.00	0.99	1.00	0.99
Adjusted *R*^2^	0.98	0.99	0.98	0.99	0.98

2 $$Y=A\times {e}^{\frac{-x}{t}}+{y}_{0}$$

where *A*, *t*, and *y*_*0*_ are correction coefficients affected by many factors, such as ice thickness, freezing temperature, initial concentration, and initial pH. *X* is the cumulative volume ratio of ice melt water, and *Y* is the concentration of Mg^2+^ in ice melt water (mg·L^−1^).

By fitting the curve, the parameter values under various conditions can be obtained, as shown in Tables 5, 6, 7 and 8. According to the experimental conditions, the values of *A*, *t*, and *y*_*0*_ are appropriately selected and substituted into the above equation to calculate the Mg^2+^ concentration of ice melt water in the melting process under specific conditions.

**Table 6:** Parameter values under different freezing temperatures

Drawing	-5℃	-10℃	-15℃	-20℃	-25℃
*y*_*0*_	22.52 ± 5.14	35.41 ± 7.70	42.72 ± 6.83	52.38 ± 6.33	60.99 ± 7.04
*A*	56.16 ± 10.28	85.67 ± 14.51	122.76 ± 23.33	157.31 ± 24.20	207.82 ± 28.08
*t*	0.36 ± 0.16	0.37 ± 0.15	0.31 ± 0.10	0.30 ± 0.07	0.30 ± 0.06
*R*^2^ (coefficient of determination)	0.99	0.99	0.99	1.00	1.00
Adjusted *R*^2^	0.97	0.97	0.98	0.99	0.99

**Table 7:** Parameter values under different initial concentrations

Drawing	300 mg·L^−1^	400 mg·L^−1^	500 mg·L^−1^	600 mg·L^−1^	700 mg·L^−1^
*y*_*0*_	21.07 ± 10.12	32.46 ± 5.82	43.15 ± 6.48	51.31 ± 4.18	61.05 ± 8.31
*A*	40.34 ± 5.53	73.57 ± 13.01	123.35 ± 23.30	146.80 ± 24.23	185.88 ± 41.55
*t*	0.55 ± 0.40	0.35 ± 0.14	0.30 ± 0.10	0.26 ± 0.06	0.28 ± 0.09
*R*^2^ (coefficient of determination)	0.98	0.99	0.99	1.00	0.99
Adjusted *R*^2^	0.95	0.97	0.98	0.99	0.98

**Table 8:** Parameter values under different initial pH values

Drawing	pH 5.5	pH 6.5	pH 7.5	pH 8.5	pH 9.5
*y*_*0*_	46.35 ± 4.65	43.15 ± 6.48	37.51 ± 14.77	39.84 ± 4.60	27.95 ± 10.90
*A*	170.63 ± 25.62	123.35 ± 23.30	90.48 ± 12.97	80.41 ± 9.22	58.405 ± 5.85
*t*	0.27 ± 0.05	0.30 ± 0.10	0.45 ± 0.26	0.36 ± 0.10	0.56 ± 0.30
*R*^2^ (coefficient of determination)	1.00	0.99	0.98	1.00	0.99
Adjusted *R*^2^	0.99	0.98	0.96	0.99	0.97

## Conclusion

- (1) In the process of water freezing, Mg^2+^ is discharged, which leads to Mg^2+^ migrating from the ice layer into the water layer under ice, and the concentration of Mg^2+^ in the water layer under ice increases. The migration effect has a more significant impact on shallow lakes at high latitudes, so more attention should be paid to the detection of Mg^2+^ pollution in the water under ice in winter.
- (2) In the water freezing process, distribution coefficient “K” decreases with increasing ice thickness, freezing temperature, initial concentration, and initial pH; in other words, higher freezing temperature, ice thickness, initial concentration, and initial pH are conducive to the migration of Mg^2+^ to the water layer under ice. Other factors (such as light and radiation) may also influence Mg^2+^ migration during the freezing process, which must be further explored.
- (3) In the early period of ice melting (0–25%), a large number of Mg^2+^ are rapidly released in a short time, and the release rate of Mg^2+^decreases rapidly in the middle and later periods (25–100%), showing a small and uniform release phenomenon. Therefore, in the early melting stage of lake ice, the preferential centralized release of pollutants may have a great impact on the ice water environment, which needs the attention of limnologists.

## Data Availability

Not applicable.
